# Long-Term Caffeine Intake Exerts Protective Effects on Intestinal Aging by Regulating Vitellogenesis and Mitochondrial Function in an Aged *Caenorhabditis Elegans* Model

**DOI:** 10.3390/nu13082517

**Published:** 2021-07-23

**Authors:** Hyemin Min, Esther Youn, Yhong-Hee Shim

**Affiliations:** Department of Bioscience and Biotechnology, Konkuk University, Seoul 05029, Korea; mintmin0701@naver.com (H.M.); dbsdptmej02@naver.com (E.Y.)

**Keywords:** caffeine, intestinal aging, anti-aging, vitellogenesis, mitochondrial function, oxidative stress response, *Caenorhabditis elegans*

## Abstract

Caffeine, a methylxanthine derived from plants, is the most widely consumed ingredient in daily life. Therefore, it is necessary to investigate the effects of caffeine intake on essential biological activities. In this study, we attempted to determine the possible anti-aging effects of long-term caffeine intake in the intestine of an aged *Caenorhabditis elegans* model. We examined changes in intestinal integrity, production of vitellogenin (VIT), and mitochondrial function after caffeine intake. To evaluate intestinal aging, actin-5 (ACT-5) mislocalization, lumenal expansion, and intestinal colonization were examined after caffeine intake, and the levels of vitellogenesis as well as the mitochondrial activity were measured. We found that the long-term caffeine intake (10 mM) in the L4-stage worms at 25 °C for 3 days suppressed ACT-5 mislocalization. Furthermore, the level of autophagy, which is normally increased in aging animals, was significantly reduced in these animals, and their mitochondrial functions improved after caffeine intake. In addition, the caffeine-ingesting aging animals showed high resistance to oxidative stress and increased the expression of antioxidant proteins. Taken together, these findings reveal that caffeine may be a potential anti-aging agent that can suppress intestinal atrophy during the progression of intestinal aging.

## 1. Introduction

The physiological effects of caffeine intake have been reported to be beneficial or harmful, depending on the dose of intake [1,2,3,4]. In general, caffeine treatment at a lower concentration (<10 mM) shows beneficial effects [3,5,6], while harmful effects are observed at higher concentrations [1,7,8]. However, we previously showed adverse effects of caffeine treatment, even at a lower concentration, on the reproduction and intergenerational effects in a *Caenorhabditis elegans* (*C. elegans)* model during the active reproductive period [4]. These findings suggested that the effects of caffeine intake are dependent not only on the caffeine concentration but also on the developmental stages of the organism when treatment is administered. Therefore, in this study, we examined the effects of caffeine intake (10 mM) during the post-developmental stage and the post-reproductive period in a *C. elegans* model.

The gonad of *C. elegans* hermaphrodites consists of germ lines containing mitotic germ cells, oocytes, and sperm, in which spermatogenesis begins at the L4 larval stage and oogenesis at the adult stage. Reproduction is the most active at day 2 of the adult stage [9,10]. In day-3 adults, fertility begins to decline, and the aging processes become active [10]. At this stage, the cost of reproduction comes at the price of oxidative stress resistance and lifespan extension [11,12,13]. *C. elegans* is an excellent animal model for studying the effects of caffeine intake on aging processes based on various indicators, including the degeneration of pharynx, presence of tumors in the uterus, atrophy of the intestine and gonads, and accumulation of lipoproteins in the body cavity [14]. Furthermore, the aged intestine of *C. elegans* showed the impaired permeability of the intestinal epithelium, small and fewer microvilli, and accumulation of the indigested food, which have also been reported in the aged intestine in mammals [15,16]. The intestinal atrophy is a prominent aging feature that is closely related to the lifespan of the animal [15,17]. Increased vitellogenesis and accumulation of the pseudocoelomic lipoprotein pool (PLP) are linked to intestinal atrophy at the advanced ages in *C. elegans* [14]. Vitellogenin (VIT) is a yolk protein that supports the development of the progeny in oviparous animals [18,19]. VITs are believed to be similar to low-density lipoproteins in humans based on their sequence similarities [20]. In *C. elegans*, VITs are exclusively synthesized in the intestine of the adult hermaphrodites; therefore, their production shows temporal-, spatial-, and sex-specificity [19,21,22]. VITs are expressed at the adult stage and continue to be produced even after the end of reproduction, and it further accelerates the aging process by increasing the intestinal atrophy [14,19]. In particular, vitellogenesis in aging *C. elegans* has been reported to be related to biomass conversion from intestine to a yolk protein by autophagic activity [14]. Interestingly, we recently reported that caffeine intake reduces VIT production during the adult stage of *C. elegans* [4]. Based on these findings, we hypothesized that caffeine intake could ameliorate intestinal atrophy, prevent intestinal aging, and eventually extend the lifespan of these aging animals by inhibiting the production of VIT. 

Aging is an irreversible phenomenon observed across different species. Studies based on the insulin/insulin-like growth factor (IGF-1) signaling in *C. elegans* aiming to reveal the mechanism underlying aging have shown that genetic factors play critical roles in the aging and regulation of the lifespan in these animals [23,24,25]. Although dietary factors are important to prevent obesity, various adult disorders and aging in addition to genetic backgrounds [26,27,28,29], the mechanisms by which the dietary factors affect intestinal aging remain elusive. In addition, our previous results showed that the intestinal atrophy was strongly observed with gliadin intake, which promoted ROS production, and the intestinal atrophy became more severe in the *mev-1* mutant that is hypersensitive to oxidative stress [30]. These findings suggest that intestinal atrophy is induced by oxidative stress. Therefore, it is important to investigate how these factors regulate the aging processes at the organism level to reveal the link among diet, intestinal aging, oxidative stress response, and lifespan of the organism, which is generally difficult to examine unless a proper animal model, such as *C. elegans*, is available. 

In this study, we attempted to examine the effects of long-term caffeine intake on the level of intestinal atrophy by evaluating the progression of intestinal aging and changes in the production of VIT, mitochondrial activity, and oxidative stress responses in the aging *C. elegans*. Here, we report that long-term caffeine intake in aging *C. elegans* reduced the VIT production, improved intestinal atrophy, promoted mitochondrial function, induced resistance against oxidative stress, and extended the life span. 

## 2. Materials and Methods

### 2.1. Caenorhabditis Elegans Strains and Treatment with Caffeine 

All strains were maintained at either 15 °C or 20 °C on the nematode growth medium (NGM) agar plates seeded with *Escherichia coli* OP50 as previously described [31]. The following strains were used in this study to analyze aging phenotype: N2 (*C. elegans* wild isolate, Bristol variety), ML2615: *dlg-1(mc103[dlg-1::GFP]) X*, for the junctional morphology [32], ERT60: *jyIs13 [act-5p::GFP::ACT-5+rol-6(su1006)] II*, for the intestinal actin localization [32], DH1033*: bIs1 (vit-2::GFP+rol-6(su10060)) X*, for the vitellogenin expression [19], DA2123: *adIs2122 [lgg-1p::GFP::lgg-1+rol-6(su1006)]*, for the autophagic activity [33], SJ4103: *zcIs14 [myo-3::GFP(mit)]*, for the muscle mitochondria, [34], SJ4143: *zcIs17 [ges-1p::GFP(mit)]*, for the intestinal mitochondria [35], CF1553*: sod-3(muls84)::GFP*, for the activity of superoxide dismutase [17], CL2166: *dvIs19 [(pAF15)gst-4p::GFP::NLS]II*, for oxidative stress response [3], LD1*: Idls7[skn-1b/c::GFP+rol-6(su1006)]*, for the stress response [36], and GR1352*: xrIs87* *[daf-16(alpha)::GFP::daf-16B+rol-6(su1006)]*, for the stress response [17].

For caffeine treatment, 10 mM caffeine (Sigma-Aldrich, St. Louis, MO, USA) was added to NGM before autoclaving, as previously described [4]. Throughout the study, we treated worms with 10 mM caffeine following our previous study showing that 10 mM of caffeine intake reduced vitellogenin production [4]. To investigate the effects of caffeine intake in aging *C. elegans*, synchronized L4 (long-term caffeine intake, caffeine treatment at 25 °C for 72 h), or 72 h post-L4 stage (short-term caffeine intake, caffeine treatment at 20 °C for 24 h) animals were examined. For the detailed experimental scheme, refer to the Supplementary Figure S1.

### 2.2. Analysis of Intestinal Aging

To analyze intestinal aging, we evaluated the pharyngeal deterioration, intestinal atrophy, localization of Actin-5 (ACT-5)::green fluorescent protein (GFP), intestinal colonization by fluorescent bacteria, and the accumulation of PLP as previously described with minor modifications [14,37]. To observe pharyngeal deterioration, discs large MAGUK scaffold protein 1 (DLG-1)::GFP transgenic animals were observed at 200× magnification under a fluorescence microscope (Zeiss Axioscope, Oberkochen, Germany). Intestinal atrophy was quantified by measuring the intestinal width at a point posterior to either the uterine tumors or vulva region, subtracting the lumenal width, and dividing it by the body width, as previously described with minor modifications [14]. To analyze the ACT-5::GFP localization in aging *C. elegans*, the ACT-5::GFP in the posterior intestine was observed at 200× magnification under a fluorescence microscope (Zeiss Axioscope, Oberkochen, Germany). To quantify the degree of OP50::GFP bacterial colonization in the intestine, synchronized L4-stage animals were fed with OP50::GFP, which is a fluorescent bacteria, on NGM plates containing 0 or 10 mM caffeine at 25 °C for 72 h. The animals were observed at 200× magnification under a fluorescence microscope (Zeiss Axioscope, Oberkochen, Germany). PLP accumulation rate was measured by the presence of the yolk pools in the body cavity at 400× magnification under a microscope (Zeiss Axioscope, Oberkochen, Germany). Between 10 and 20 worms were observed for each set of DLG-1::GFP, OP50::GFP, and PLP accumulation. For intestinal width measure, a total of 24 worms were observed.

### 2.3. Live Image Observation of Fluorescence-Tagged Transgenic Animals

To observe the gene expression of each transgenic animal, synchronized L4-stage animals were fed 0 or 10 mM caffeine at 25 °C for 72 h. Then, the animals were mounted on a poly-L-lysine (Sigma-Aldrich, St. Louis, MO, USA) coated glass slide using 10 µL M9 containing 0.2 mM tetramisole hydrochloride (Sigma-Aldrich, St. Louis, MO, USA). Live worm images were acquired under a fluorescence microscope (Zeiss Axioscope, Oberkochen, Germany) and processed using the Nikon NIS-Elements Basic Research imaging software v.4.3. The fluorescence intensity was quantified using the ImageJ software. Between 10 and 20 worms were observed with fluorescence-tagged transgenic animals for each set of experiments except for VIT-2::GFP observation. A total of 27 worms were observed with VIT-2::GFP transgenic animals under the condition of 0 mM caffeine treatment. 

### 2.4. Western Blot Analysis

Western blot analysis was performed as described previously [4]. The DH1033: *bIs1 (vit-2::GFP+rol-6(su10060))* transgenic animal protein extract prepared from 30 adult hermaphrodites of each treatment group was subjected to sodium dodecyl sulfate–polyacrylamide gel electrophoresis and transferred to nitrocellulose membrane. Antibodies bound to a nitrocellulose membrane (PROTRAN BA83, Whatman; Sigma-Aldrich, St. Louis, MO, USA) were visualized using an ECL Western blotting detection kit (Amersham, GE Healthcare Life Sciences, Pittsburgh, PA, USA), and the band intensities were measured using a LAS-3000 image analyzer equipped with Multi Gauge v.3.0 (Fuji Film, Tokyo, Japan). The following primary and secondary antibodies were used: rabbit anti-GFP (1:1000; Novus Biologicals, Centennial, CO, USA), mouse anti-α-tubulin (1:1000; Sigma-Aldrich, St. Louis, MO, USA), horseradish peroxidase (HRP)-conjugated goat anti-rabbit IgG (1:1000; Santa Cruz Biotechnology, Dallas, TX, USA), and HRP-conjugated donkey anti-mouse IgG (1:1000; Jackson ImmunoResearch, West Grove, PA, USA).

### 2.5. Quantitative Reverse Transcription-Polymerase Chain Reaction (qRT-PCR)

Real-time qRT-PCR was performed as described previously [4]. Briefly, RNA was isolated from adult hermaphrodites treated with 0 or 10 mM caffeine at the L4 stage at 25 °C for 72 h. The animals were placed in the TRIzol reagent (Invitrogen, Waltham, MA, USA), and total RNA was extracted using standard phenol–chloroform extraction and ethanol precipitation method using a phase lock gel (MaXtract High Density; Qiagen, Germantown, MD, USA) with 150 adult hermaphrodites of each treatment group. cDNA was synthesized using oligo-dT primers and the Moloney Murine Leukemia Virus (M-MLV) reverse transcriptase (Invitrogen, Waltham, MA, USA). qRT-PCR assays were performed on ABI 7500 (Applied Biosystems, Waltham, MA, USA) using SYBR Green PCR Master Mix (Applied Biosystems, Waltham, MA, USA). The final PCR volume was 10 µL, with 50 ng of the converted cDNA. The *act-1* mRNA was used as an endogenous control for the normalization of data. The primers used to measure the expression levels of each gene were as follows: *act-1* forward, 5′-CCAGGAATTGCTGATCGTATGCAGAA-′3; *act-1* reverse, 5′-TGGAGAGGGAAGCGAGGATAG-3; *unc-62* forward, 5′-TAAGACATACCCAAGAGAATGCTG-′3; *unc-62* reverse, 5′-TTTGCCTTTCAGACAGACCA-′3; *ceh-60* forward, 5′-AGTTCTACGGTTGCATCTTCG-′3; *ceh-60* reverse, 5′-AGTGTGGCTGATGGAGAAAC-′3; *pqm-1* forward, 5′-TCTCGAAAATGTCCGCACTG-3′; *pqm-1* reverse, 5′-GAGGTTCTTTCACGAATTGCTTC-3′. The GenBank database accession number for *C. elegans* mRNA and the product size of each qRT-PCR are: *act-1* (NC_003283.11, Chr V: 11081052..11082415): 133 bp, *unc-62* (NC_003283.11, Chr V: 4497463..4511447): 133 bp, *ceh-60* (NC_003284.9, Chr X: 6522596..6530146): 148 bp, *pqm-1* (NC_003280.10, Chr II: 11150391..11152595): 125 bp.

### 2.6. Analysis of Reactive Oxygen Species (ROS) Production in Mitochondria

To examine the effect of caffeine intake on the mitochondrial ROS, CellROX^®^ Green (Invitrogen, Carlsbad, CA, USA) staining was performed as described previously [4,38]. Briefly, CellROX^®^ Green was freshly prepared as 5 mM stock solutions and diluted in the M9 buffer at a 1:500 dilution before treatment. Then, the animals were transferred to either 0 or 10 mM caffeine plates containing the staining solution and stained at 20 °C for 2 h. The animals were mounted on a poly L-lysine-coated slide and observed under a fluorescence microscope (Zeiss Axioscope, Oberkochen, Germany). The relative quantification of mitochondrial ROS was performed using the ImageJ software. More than 15 worms were observed for each set of the experiments.

### 2.7. Analysis of the Mitochondrial Membrane Potential (MMP) 

To measure MMP, tetramethylrhodamine methyl ester (TMRM; Thermo Fisher Scientific, Waltham, MA, USA) staining was performed as previously described [38]. Briefly, TMRM (final concentration: 30 µM) was added to NGM agar plates containing 0 or 10 mM caffeine. The plates were then seeded with dead *E. coli* OP50, and dried for 24 h in the dark. The synchronized animals were transferred to TMRM plates and incubated at 20 °C for 15 h. Then, the animals were mounted on a poly L-lysine-coated slide and observed under a fluorescence microscope (Zeiss Axioscope, Oberkochen, Germany). Fluorescence intensity was measured using the ImageJ software. More than 10 worms were observed for each set of the experiments except the young adult with 0 mM caffeine treatment in which 12 worms were observed.

### 2.8. Motility Assay

To analyze the effect of long-term caffeine intake on motility, body bends were measured as previously described [38]. Briefly, animals from each test condition were transferred onto separate NGM plates and scored for the number of body bends at 20 s intervals. One body bend was defined as a complete cycle of terminal bulb motion, starting from the top position of the sinusoidal wave track through to the bottom and back to the top. A total of 21 worms were observed for each treatment.

### 2.9. Survival Assay

The survival assay was performed as previously described [39]. Synchronized animals were transferred to NGM plates containing 0 or 10 mM caffeine. Following treatment, the plates were observed daily under a dissecting microscope, and the animal viability was scored. The animals were judged to be dead if they did not respond to gentle poking with a platinum wire. Percent survival was calculated as the percentage of surviving animals in the population. A total of 20 worms were observed for each treatment.

### 2.10. Survival Assay under Paraquat-Induced Oxidative Stress

The paraquat survival assay was performed as previously described, with minor modifications [38]. To analyze the survival rates under oxidative stress conditions in each group, the synchronized 72 h post-L4-stage animals were exposed to 100 mM paraquat solution at 20 °C for 3 h, and subsequently, the number of dead and live animals were counted. The animals were considered dead when they failed to respond to a gentle touch with a platinum wire on their bodies. More than 30 worms were observed for each treatment. 

### 2.11. Statistical Analysis

All experiments were repeated more than three times for statistical evaluation of the data. The *p* values were calculated using either a two-tailed Student’s *t*-test or one-way analysis of variance (ANOVA) with Tukey’s post hoc test. Statistical significance was set at *p* < 0.05. Data are expressed as the mean ± standard deviation (SD). Statistical analyses were performed using the jamovi software (https://www.jamovi.org/, accessed on 13 November 2020).

## 3. Results

### 3.1. Long-Term Caffeine Intake Prevents Intestinal Aging in Caenorhabditis elegans 

One of the mechanisms underlying intestinal aging was recently identified, demonstrating that vitellogenesis is coupled to intestinal atrophy mediated by autophagy, which facilitates intestinal biomass conversion to sustain vitellogenin synthesis in aging *C. elegans* [14]. In our previous study, caffeine intake was shown to reduce vitellogenin production in *C. elegans* [4]. Here, we investigated whether reduced vitellogenesis caused by caffeine intake affects the progression of intestinal aging in *C. elegans*. To determine the effect of caffeine intake on intestinal aging, we examined intestinal atrophy after short-term and long-term caffeine intake in aging wild-type *C. elegans* (Supplementary Figures S1 and S2). We found that both short-term and long-term caffeine ingestion in these animals significantly improved intestinal atrophy compared to the 0 mM caffeine aging group (Supplementary Figure S2). However, among the caffeine groups, long-term caffeine intake was the most effective in preventing intestinal atrophy during aging (Supplementary Figure S2). These results suggest that caffeine intake exerts a preventive effect on intestinal aging, and long-term caffeine intake is particularly effective in aging *C. elegans*. Therefore, to further understand how caffeine intake effectively protects against intestinal aging, we investigated long-term caffeine intake at 25 °C, which accelerates aging in *C. elegans* (Supplementary Figure S1). 

**Figure 1:**
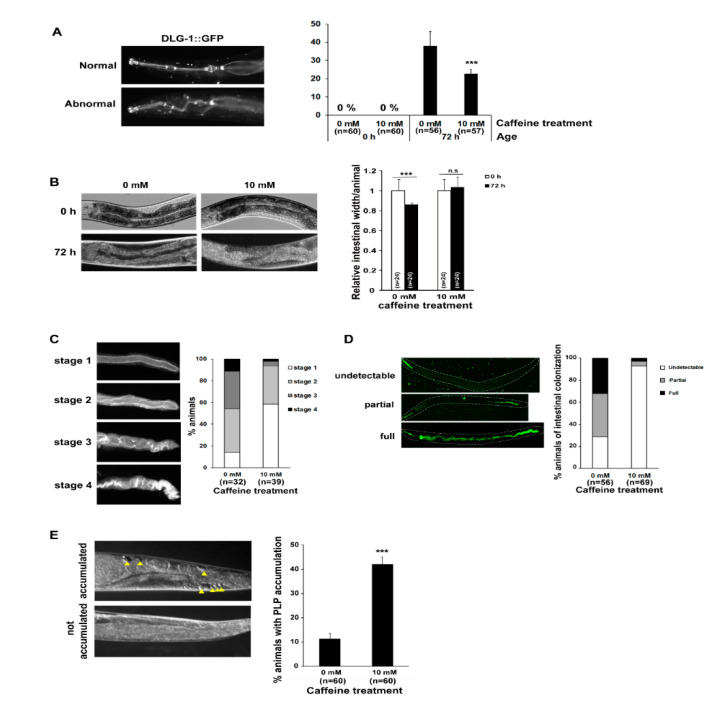
Long-term caffeine intake delays intestinal aging during advanced ages of *Caenorhabditis elegans*: (**A**) Animals expressing discs large MAGUK scaffold protein 1 (DLG-1):: green fluorescent protein (GFP) were treated with 0 or 10 mM caffeine at the L4 stage at 25 °C for 72 h. The location of DLG-1::GFP in the pharynx was observed at 72 h post-L4 stage at 25 °C. Error bars represent standard deviation (SD). *** *p* < 0.001 (one-way analysis of variance (ANOVA) with Tukey’s post hoc test); (**B**) The synchronized wild-type L4-stage animals were treated with 0 or 10 mM caffeine at 25 °C for 72 h. The intestinal atrophy was measured at 72 h post-L4 stage at 25 °C. Error bars represent SD. *** *p* < 0.001. n.s., not significant (two-way ANOVA with Tukey’s post hoc test); (**C**) Animals expressing actin 5 (ACT-5)::GFP were treated with 0 or 10 mM caffeine at the L4 stage at 25 °C for 72 h. The type of mislocalization was classified into four stages. The percent distributions of the respective stages in animals fed with 0 or 10 mM caffeine are presented; (**D**) The synchronized wild-type L4-stage animals were fed with *E. coli* OP50::GFP, a fluorescent bacteria on 0 or 10 mM caffeine nematode growth medium (NGM) plates at 25 °C for 72 h. The type of bacterial colonization was classified into three categories: (1) undetectable, (2) partial, and (3) full. The percent distributions of the respective categories in animals treated with 0 or 10 mM caffeine are presented; (**E**) The synchronized wild-type L4-stage animals were treated with 0 or 10 mM caffeine at 25 °C for 72 h. Accumulation of the pseudocoelomic lipoprotein pool (PLP) (indicated by yellow arrowheads) was observed at 72 h in post-L4-stage animals at 25 °C. The percentage of animals with PLP accumulation among the total number of animals is shown. Error bars represent SD. *** *p* < 0.001 (*t*-test).

We investigated the effects of long-term caffeine intake on intestinal aging phenotypes, including pharyngeal deterioration and intestinal atrophy, based on a previous report [14], (Figure 1). Pharyngeal deterioration was analyzed by observing a GFP-tagged *dlg-1* transgene to determine the integrity of epithelial junctional localization with age [32,40]. We found that caffeine-ingested animals showed a significantly reduced pharyngeal deterioration with age compared to the animals subjected to the caffeine-free diet (Figure 1A). In particular, severe intestinal atrophy developed with age in caffeine-free diet animals, whereas caffeine-ingested animals showed significantly decreased intestinal atrophy with age (Figure 1B). The mislocalization of ACT-5, indicating the disrupted integrity of the *C. elegans* intestinal barrier and accelerated pathogenesis with age [32], was also confirmed by observing ACT-5::GFP transgenic animals after caffeine intake (Figure 1C). Consistent with the results of ACT-5 mislocalization, a caffeine-free diet in aging animals promoted intestinal colonization of GFP-expressing *E. coli*, as detected by fluorescence microscopy (Figure 1D). However, caffeine-ingested aging animals showed less bacterial colonization in the intestine compared to caffeine-free diet animals (Figure 1D). Moreover, PLP accumulation, which is closely correlated with intestinal atrophy [14], significantly decreased following caffeine intake in aging animals (Figure 1E). These results suggest that long-term caffeine intake prevents intestinal aging by maintaining intestinal integrity in aging *C. elegans*. 

### 3.2. Long-Term Caffeine Intake Reduces Vitellogenesis in Aging Caenorhabditis elegans 

**Figure 2:**
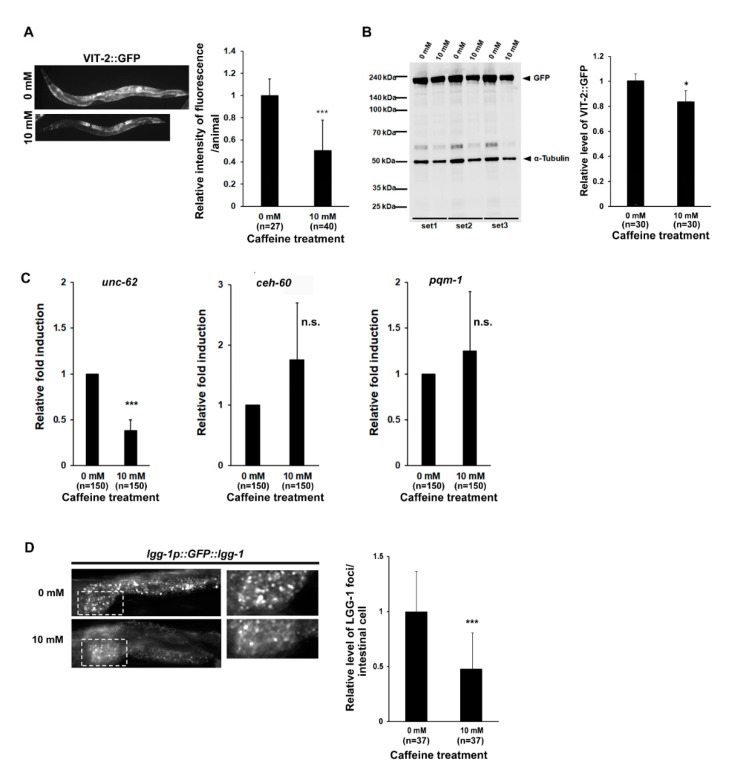
Long-term caffeine intake decreases vitellogenesis in advanced ages of *C. elegans*: (**A**) Animals expressing vitellogenin (VIT)-2::GFP were treated with 0 or 10 mM caffeine at the L4 stage at 25 °C for 72 h. The expression of VIT-2::GFP was observed at 72 h in post-L4-stage animals at 25 °C. Error bars represent SD. *** *p* < 0.001 (*t*-test); (**B**) Western blot analysis of VIT-2::GFP protein levels using an anti-GFP antibody in each test condition. α-tubulin was used as the loading control. The relative expression levels of GFP in each condition are shown. GFP band intensity was normalized to that of α-tubulin on the same lane, and the relative levels of GFP were converted to a relative value against that of the animals fed with 0 mM caffeine as 1. Error bars represent SD. * *p* < 0.05 (*t*-test); (**C**) The synchronized wild-type L4-stage animals were treated with 0 or 10 mM caffeine at 25 °C for 72 h. The mRNA levels of *unc-62*, *ceh-60*, and *pqm-1* in the caffeine-free or caffeine-ingested animals were determined by three independent quantitative reverse transcription-polymerase chain reaction (qRT-PCR) tests using the mRNA level of *act-1* in each sample as an internal control for normalization. Error bars represent SD. *** *p* < 0.001. n.s., not significant (*t*-test); (**D**) Animals expressing *lgg-1p::GFP::lgg-1* were treated with 0 or 10 mM caffeine at the L4 stage at 25 °C for 72 h; then, we imaged GFP::LGG-1 foci in the intestinal cells at 72 h post-L4-stage animals at 25 °C. The white dotted box represents the GFP::LGG-1 foci in the intestinal cells, and the right panel shows an enlarged image. The graph shows the relative levels of GFP::LGG-1 foci in the intestinal cells after caffeine treatment. Error bars represent SD. *** *p* < 0.001. n.s., not significant (*t*-test).

How does long-term caffeine intake prevent intestinal aging? It has been shown that *C. elegans* consumes its own intestine via autophagy to produce VIT and biomass conversion during advanced ages [14]. Notably, caffeine intake can reduce the VIT production at the adult stage in *C. elegans* [4]. These observations suggest that caffeine intake modulates intestinal aging by reducing VIT production. To test this hypothesis, we analyzed the level of expression of VIT-2::GFP using transgenic animals by both fluorescence microscopic observation and Western blotting after long-term caffeine treatment (Figure 2A,B). The amount of VIT-2 protein in response to long-term caffeine intake reduced to less than 0.5 fold, as observed by fluorescence microscopy. Quantitative analysis of VIT-2::GFP protein by Western blotting showed that its expression decreased by approximately 80% compared to that in the caffeine-free diet animals (Figure 2A,B). These results suggest that the reduced vitellogenin production in response to caffeine intake prevents intestinal aging in the advanced age of *C. elegans*.

Some transcriptional regulators of vitellogenesis, such as *unc-62*, *ceh-60*, and *pqm-1*, have been identified [41,42]. To explore whether these transcription regulators control vitellogenesis, we measured the expression levels of *unc-62*, *ceh-60*, and *pqm-1* using qRT-PCR analysis after caffeine treatment (Figure 2C). Caffeine-ingested animals showed a 0.38 (±0.12)-fold decline in the expression of *unc-62* in aging animals as compared to that in the caffeine-free diet animals (Figure 2C), suggesting that the reduction of VIT-2 level in response to caffeine intake is due to the low level of *unc-62*, which is consistent with the fact that *unc-62* is a transcriptional activator of *vit-2* [4]. This finding also suggests that *unc-62* is the major regulator of vitellogenesis in caffeine-treated animals.

Next, we verified whether the previously reported autophagy activity, which plays a major role in promoting intestinal biomass conversion for vitellogenesis, is reduced by caffeine intake in aging animals. We observed autophagy activity using *lgg-1p::GFP::lgg-1* transgenic animals subjected to fluorescence microscopy after long-term caffeine treatment. Caffeine-ingested animals showed a 0.47 (±0.32)-fold decline in the level of LGG-1 foci in the intestinal cell compared to that in the caffeine-free diet animals (Figure 2D). Taken together, these results suggest that long-term caffeine intake decreases vitellogenesis by reducing *unc-62* expression and intestinal autophagy activity, which prevents intestinal atrophy in advanced age.

### 3.3. Long-Term Caffeine Intake Promotes Mitochondrial Function in Aging Caenorhabditis elegans 

**Figure 3:**
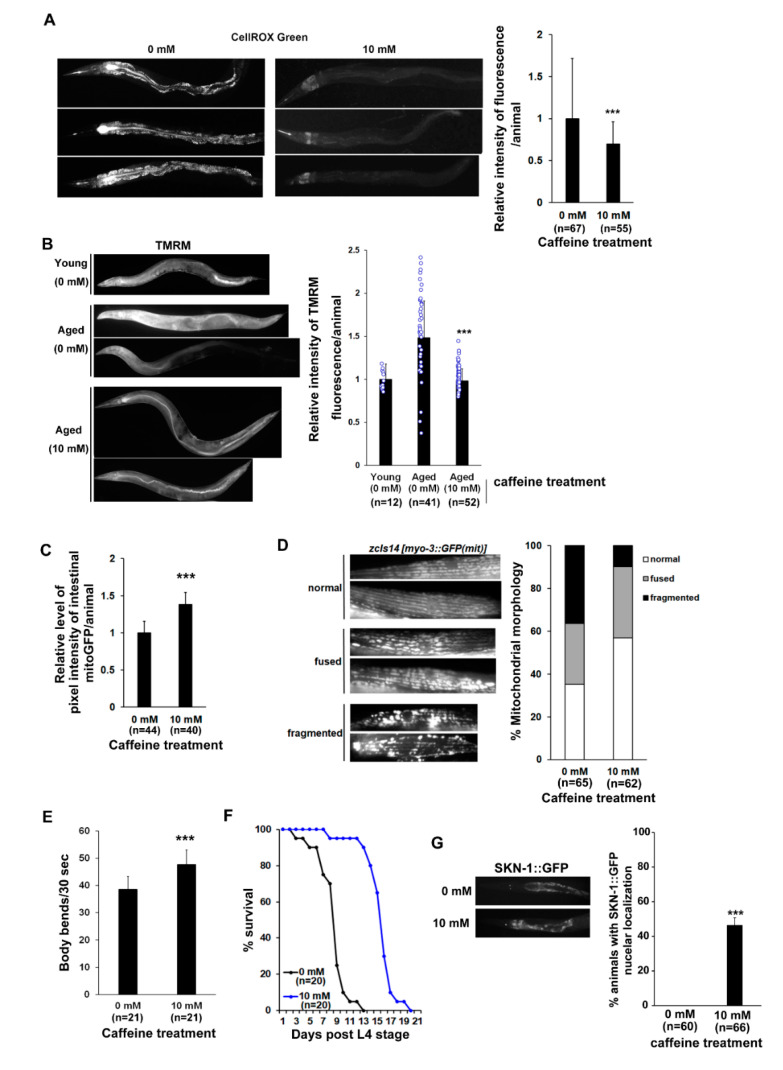
Long-term caffeine intake improves mitochondrial function and motility during advanced ages of *C. elegans*: (**A**) Comparison of the mitochondrial reactive oxygen species (ROS) levels between the caffeine-free and caffeine-ingested animals by CellROX Green staining. Wild-type animals were treated with 0 or 10 mM caffeine at the L4 stage at 25 °C for 72 h. The graph shows the relative levels of mitochondrial ROS analyzed by ImageJ. Error bars represent SD. *** *p* < 0.001 (*t*-test); (**B**) Comparison of the mitochondrial membrane potential (MMP) in young (0 mM) and aged (0 mM; 10 mM) animals using tetramethylrhodamine methyl ester (TMRM) staining. In the aged groups, wild-type animals were treated with 0 or 10 mM caffeine at the L4 stage at 25 °C for 72 h. In the young (0 mM) group, wild-type animals were treated with 0 mM caffeine at the L4 stage at 25 °C for 24 h. The TMRM fluorescence was quantified for each test condition by ImageJ. The graph shows the relative levels of MMP. Error bars represent SD. *** *p* < 0.001 (one-way ANOVA with Tukey’s post hoc test); (**C**) Comparison of intestinal mitochondrial activity in *ges-1p::GFP(mit)* transgenic animal expressing *ges-1* promoter driven GFP. The transgenic animals were treated with 0 or 10 mM caffeine at the L4 stage at 25 °C for 72 h. The graph shows the relative levels of fluorescence intensity as analyzed by ImageJ. Error bars represent SD. *** *p* < 0.001 (*t*-test); (**D**) The mitochondrial morphology was analyzed using the SJ4103 transgenic animal expressing a mitochondrial-targeted GFP under the control of the muscle-specific *myo-3* promoter. The transgenic animals were treated with 0 or 10 mM caffeine at the L4 stage at 25 °C for 72 h. The graph indicates the percentage of animals with muscle mitochondria classified into three categories: (1) normal, (2) fused, and (3) fragmented; (**E**) Comparison of body bending in caffeine-free diet animals and caffeine-ingested animals at advanced ages. Wild-type animals were treated with 0 or 10 mM caffeine at the L4 stage at 25 °C for 72 h. Error bars represent SD. *** *p* < 0.001 (*t*-test); (**F**) Comparison of the survival rates between the caffeine-free diet animals and caffeine-ingested animals. Wild-type animals were treated with 0 or 10 mM caffeine at the L4 stage until dead at 25 °C; (**G**) Animals expressing skinhead 1 (SKN-1)::GFP were treated with 0 or 10 mM caffeine at the L4 stage at 25 °C for 72 h. The expression levels of SKN-1::GFP were observed at 72 h post-L4-stage animals at 25 °C. Error bars represent SD. *** *p* < 0.001 (*t*-test).

It has been suggested that immunity and antioxidant defense, which regulate ROS production in response to bacterial colonization during intestinal aging, are major effectors of aging and lifespan in *C. elegans* [43,44]. Given that caffeine intake suppresses bacterial colonization of the intestine in aging animals (Figure 1D), we measured mitochondrial ROS levels using CellROX Green, which is a fluorogenic probe for measuring mitochondrial ROS in live cells [38]. We observed that the fluorescence intensity decreased significantly in the long-term caffeine-ingested wild-type animals (Figure 3A). In addition, long-term caffeine-ingested animals showed normal MMP (Figure 3B). Mitochondrial activity was increased in the intestine of intestinal mitoGFP transgenic animals compared to that in the caffeine-free diet animals of advanced age (Figure 3C). These results suggest that long-term caffeine intake inhibits bacterial colonization and maintains normal mitochondrial function, resulting in a low level of ROS production at advanced ages.

To further investigate the modulations in mitochondria in response to caffeine intake at advanced ages, we examined morphological changes in the mitochondria using *myo-3p::mitoGFP* transgenic animals, which are expressed in the mitochondria of the muscle cells. The caffeine-free diet animals at advanced ages showed increased altered muscle mitochondrial morphology, including ‘fused (28.23%)’ and ‘fragmented (39.53%)’ mitochondria, while the caffeine-ingested animals in advanced ages exhibited a decreased ‘fragmented (9.93%)’ muscle mitochondrial morphology and an increased normal morphology (56.83%) (Figure 3D). This result indicates that long-term caffeine intake improves the integrity of mitochondrial morphology in advanced age groups.

Since abnormal mitochondria in muscle cells are associated with changes in locomotion behavior [45], we tested whether long-term caffeine intake affects motility by measuring the body bending rates at advanced ages. Compared to the caffeine-free diet animals, the body bending rate increased significantly in the caffeine-treated animals at advanced ages (Figure 3E). Consistent with the correlation between motility activity and lifespan [46], we also confirmed that long-term caffeine intake increased the survival rate (Figure 3F). Moreover, *skn-1* activation was evaluated, since it is the primary target to be activated by nuclear localization for regulating the survival rate [47,48]. In our study, SKN-1 was activated in the intestine after caffeine treatment (Figure 3F,G), implying that long-term caffeine intake promotes motility and extends the lifespan mediated by SKN-1 activation.

### 3.4. Long-Term Caffeine Intake Induces Oxidative Stress Response in Aging Caenorhabditis elegans 

**Figure 4:**
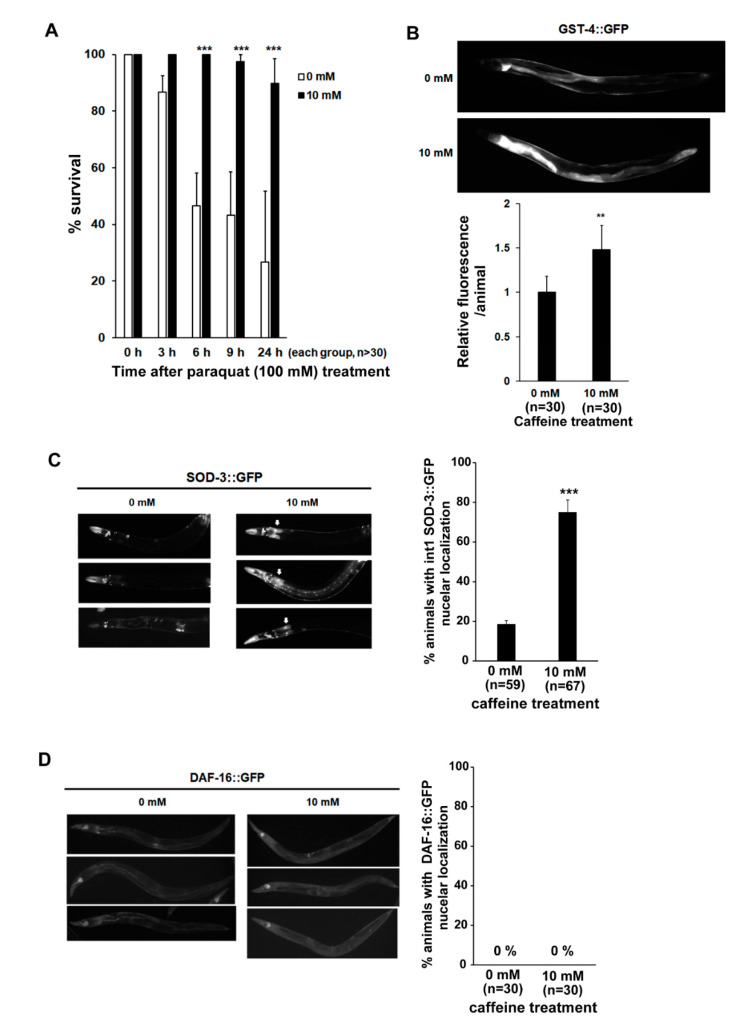
Long-term caffeine intake exerts a protective effect on oxidative stress in advanced ages of *C. elegans*: (**A**) Percentage survival rates of caffeine-free diet and caffeine-ingested animals were analyzed under paraquat (100 mM)-induced oxidative stress condition. Error bars represent SD. *** *p* < 0.001 (two-way ANOVA with Tukey’s post hoc test); (**B**) Animals expressing glutathione S-transferase 4 (GST-4)::GFP were treated with 0 or 10 mM caffeine at the L4 stage at 25 °C for 72 h. The expression of GST-4::GFP was observed at 72 h post-L4-stage animals at 25 °C. The graph shows the relative fluorescence intensity analyzed by ImageJ. Error bars represent SD. ** *p* < 0.01 (*t*-test); (**C**) Animals expressing superoxide dismutase 3 (SOD-3)::GFP were treated with 0 or 10 mM caffeine at the L4 stage at 25 °C for 72 h. The expression levels of SOD-3::GFP were observed at 72 h post-L4-stage animals at 25 °C. The graph indicates the percentage of animals with SOD-3::GFP nuclear localization in the intestinal cells. Error bars represent SD. *** *p* < 0.001 (*t*-test); (**D**) Animals expressing DAF-16::GFP were treated with 0 or 10 mM caffeine at the L4 stage at 25 °C for 72 h. The expression levels of DAF-16::GFP were observed at 72 h post-L4-stage animals at 25 °C. The graph indicates the percentage of animals with DAF-16::GFP nuclear localization in the intestinal cells.

Next, we examined whether long-term caffeine intake alters the sensitivity of oxidative stress response in animals at advanced ages. Caffeine-ingested animals showed a significantly higher survival rate compared to the caffeine-free diet animals upon exposure to 100 mM paraquat, indicating that caffeine intake promotes resistance to oxidative stress in advanced ages (Figure 4A).

The SKN-1-mediated oxidative stress response involves the upregulated expression of phase II detoxification enzymes, such as GST-4 [36]. DAF-16 is a forkhead box O (FOXO) transcription factor that responds to various stresses. Superoxide dismutase 3 (SOD-3), an antioxidant enzyme, is one of the transcriptional targets of DAF-16 [17]. Therefore, we hypothesized that the resistance to oxidative stress mediated by caffeine intake may result from the changes in the expression levels of factors that regulate the transcription of essential detoxification genes, such as *gst-4* and *sod-3* [49,50,51]. Indeed, we found that the activation of GST-4::GFP was significantly increased in caffeine-treated animals, suggesting that caffeine intake induces the detoxification enzyme GST-4 via SKN-1 activation to promote resistance to oxidative stress (Figure 3G and Figure 4B). We also found a significant increase in SOD-3::GFP in caffeine-treated animals at advanced ages (Figure 4C). However, contrary to our expectation, DAF-16 was not activated by long-term caffeine intake in the advanced age groups (Figure 4D), although both SKN-1 and DAF-16 were activated by heat treatment (Supplementary Figure S3). Collectively, these results suggest that long-term caffeine intake increases GST-4 dependent SKN-1 activity and promotes SOD-3 activity in a manner distinct from that of DAF-16.

## 4. Discussion

To examine the effects of caffeine intake on aging in somatic tissues, we first investigated the intestinal aging of *C. elegans*, since the most significant changes related to aging were documented in the intestine. In addition, *C. elegans* is an excellent animal model for evaluating the effects of long-term nutrient intake due to its short life span, which is further accelerated by growth at higher temperatures (25 °C). Therefore, in this study, we explored the effects of long-term caffeine intake on intestinal aging during adulthood. Here, we showed that long-term caffeine intake during adulthood prevented intestinal atrophy and dysfunction in aging *C. elegans*. We demonstrated that the intestinal aging phenotypes, including pharyngeal deterioration, intestinal atrophy, ACT-5 mislocalization, bacterial colonization, VIT production, and PLP accumulation were significantly suppressed by long-term caffeine intake. Our study also provides evidence that long-term caffeine intake contributes to the maintenance of redox homeostasis and mitochondrial function, including the membrane potential, activity, and morphology of mitochondria in the aging animals. Furthermore, long-term caffeine intake significantly improves the motility and lifespan of these organisms. These findings indicate that long-term caffeine intake at a low dose (10 mM) during adulthood contributes to improving the integrity of the intestine as well as the mitochondrial function in advanced ages. Although the effects of long-term use of caffeine for the elderly are still controversial with both positive and adverse effects in human [52,53,54], our results obtained from the *C. elegans* model showed positive effects on the intestinal aging. 

Our results revealed that long-term caffeine intake prevents intestinal aging via regulating vitellogenesis in aging *C. elegans*, suggesting that long-term dietary habits affect the regulation of lipoprotein production, which is linked to aging. Based on our results, we propose a mechanism by which the suppression of vitellogenesis in long-term caffeine intake is regulated by *unc-62*, which is an essential transcriptional factor for the expression of VIT in *C. elegans* [55]. It was previously reported that *unc-62* RNAi increased the expression of SOD-3::GFP in aged animals and extended the lifespan of *C. elegans* [55]. It was also shown that autophagy promotes intestinal atrophy and yolk steatosis [14]. Therefore, we speculate that the decrease in *unc-62* expression due to long-term caffeine intake may suppress vitellogenesis and contribute to intestinal integrity by inhibiting the autophagy, improving the mitochondrial function and redox homeostasis, and extending the lifespan of the organism. It is necessary to further investigate the effects of inhibiting the expression of genes involved in autophagy or *unc-62* activity on intestinal aging, mitochondrial function, and oxidative stress responses in aging organisms. The *C. elegans* VIT proteins contain domains homologous to apoB-100, which is the apoprotein of the low-density lipoprotein (LDL) in humans [20]. VIT proteins bind to and transport lipids, such as triglycerides and cholesterol, to oocytes, thereby showing a similar function to the LDL in mammals [56]. In *C. elegans*, a mechanism causing intestinal atrophy-mediated bioconversion (via intestinal autophagy) for yolk synthesis has been identified [14]. Apart from autophagy, this intestine-to-yolk biomass conversion is also mediated by insulin/insulin-like growth factor (IGF-1) signaling [14]. Loss of function of genes activating autophagy suppresses intestinal atrophy, indicating that autophagy facilitates intestine-to-yolk biomass conversion, and vitellogenesis plays a crucial role in intestinal atrophy in aging *C. elegans.* In humans, hyperlipidemia occurs with advanced age; in particular, LDL hypercholesterolemia plays a causative role in the pathogenesis of cardiovascular diseases, indicating that the levels of cholesterol-rich LDL and other apolipoprotein B (apoB)-containing lipoproteins are directly implicated in the development of cardiovascular diseases [57,58]. In terms of hyperlipidemia, enhanced vitellogenesis in advanced ages of *C. elegans* imitates this condition. Therefore, identifying the mechanism underlying the regulation of lipoprotein production during aging is imperative in understanding the basis of the aging. In particular, the discovery of dietary lipoprotein regulators related to aging, such as vitellogenin, is important because of its close relevance to humans.

Mitochondrial alterations lead to a decline in energy production at the cellular level, which is associated with aging, as shown in animal models as well as human tissues [45,59,60,61,62]. The human colon is markedly affected by the progression of mitochondrial aging, which has emerged as an important player in intestinal tissue homeostasis and pathogenesis [63]. An association between aging human colonic cells and defective complexes of the respiratory chain has also been described [59]. Our findings showed a strong association between intestinal integrity and mitochondrial function in response to long-term caffeine intake in aging *C. elegans*. This speculation is supported by a recent report, which showed that mitochondrial dysfunction with increased reactive oxygen species production is a potential cause of intestinal aging [64]. Furthermore, inflammation along with mitochondrial dysfunction is a major pathological factor for the intestinal atrophy [65]. Therefore, anti-inflammatory effects of caffeine intake remain to be determined. In our study, long-term caffeine intake increased the expression of essential detoxification genes, including *gst-4* and *sod*-3, which were accompanied by SKN-1 activity, but not DAF-16, suggesting that the effects of long-term caffeine intake in aging animals are associated with SKN-1 activity but are independent of DAF-16 activity. However, the possibility of short-term induction of DAF-16, which was not sustained at the point of observation in the long-term caffeine intake condition, cannot be ruled out. This notion is supported by previous observations that DAF-16 is activated by short-term caffeine intake and is also temporally activated by probiotic microorganisms in *C. elegans* [4,66]. DAF-16 is a FOXO transcription factor that responds to various stresses, and one of the transcriptional targets of DAF-16 is the *sod-3* encoding the antioxidant enzyme SOD-3 [17].

**Figure 5:**
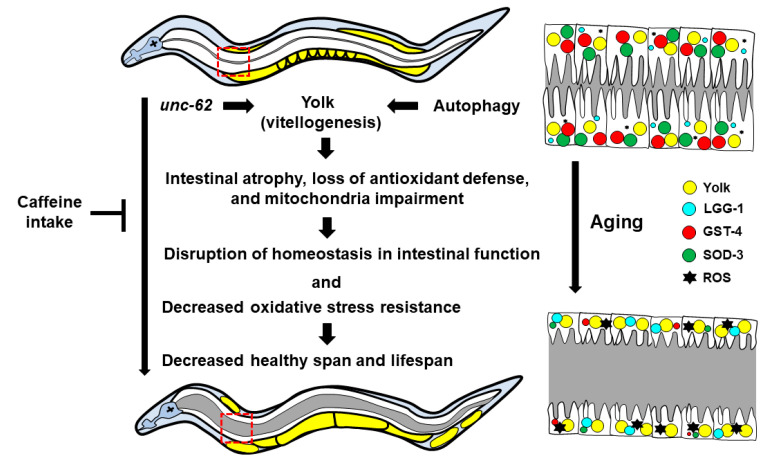
A working model to explain the protective effects of long-term caffeine intake on intestinal aging *C. elegans* at advanced ages. Long-term caffeine intake reduces vitellogenesis via regulating the expression of *unc-62* and autophagy. The decrease in vitellogenesis in response to caffeine intake delays the intestinal atrophy along with improving the mitochondrial function and antioxidant defense. Maintaining homeostasis of intestinal function via caffeine intake supports the health and lifespan in *C. elegans* at advanced ages.

Based on our study outcomes, we propose a model for the regulation of vitellogenesis via long-term caffeine intake (Figure 5), thereby providing a possible molecular mechanism that links intestinal aging, mitochondrial function, and health in the context of diet-induced regulation of aging. We also propose that intestinal integrity and mitochondrial functions are closely interconnected, and that “what to eat on a long-term basis” and “when to eat” are important factors regulating the aging process. Furthermore, caffeine and related purine alkaloids, such as theophylline and theobromine, have been reported to exhibit pro-oxidant and lifespan extension effects in *C. elegans* at low concentrations (5 mM) [3]. It will be interesting to study whether other caffeine-analogs also exhibit similar effects on the intestinal aging and mitochondrial function in *C. elegans*.

## Supplementary Materials

The following are available online at https://www.mdpi.com/article/10.3390/nu13082517/s1, Figure S1: Scheme of the assay used to determine the effects of caffeine intake on intestinal aging, Figure S2: Effects of the caffeine exposure duration on intestinal atrophy, Figure S3: Effects of heat shock on the localization of skinhead 1 (SKN-1)::green fluorescent protein (GFP) and DAF-16::GFP in *Caenorhabditis elegans*.
