# Supplementary material for: Long-Term Caffeine Intake Exerts Protective Effects on Intestinal Aging by Regulating Vitellogenesis and Mitochondrial Function in an Aged Caenorhabditis Elegans Model
Source: Nutrients. 2021 Jul 23;13(8):2517. doi: 10.3390/nu13082517 (PMC8398797; doi:10.3390/nu13082517)
Supplement: Supplementary file 1 [file nutrients-13-02517-s001.zip › nutrients-1286081-supplementary.pptx]

## Slide 1
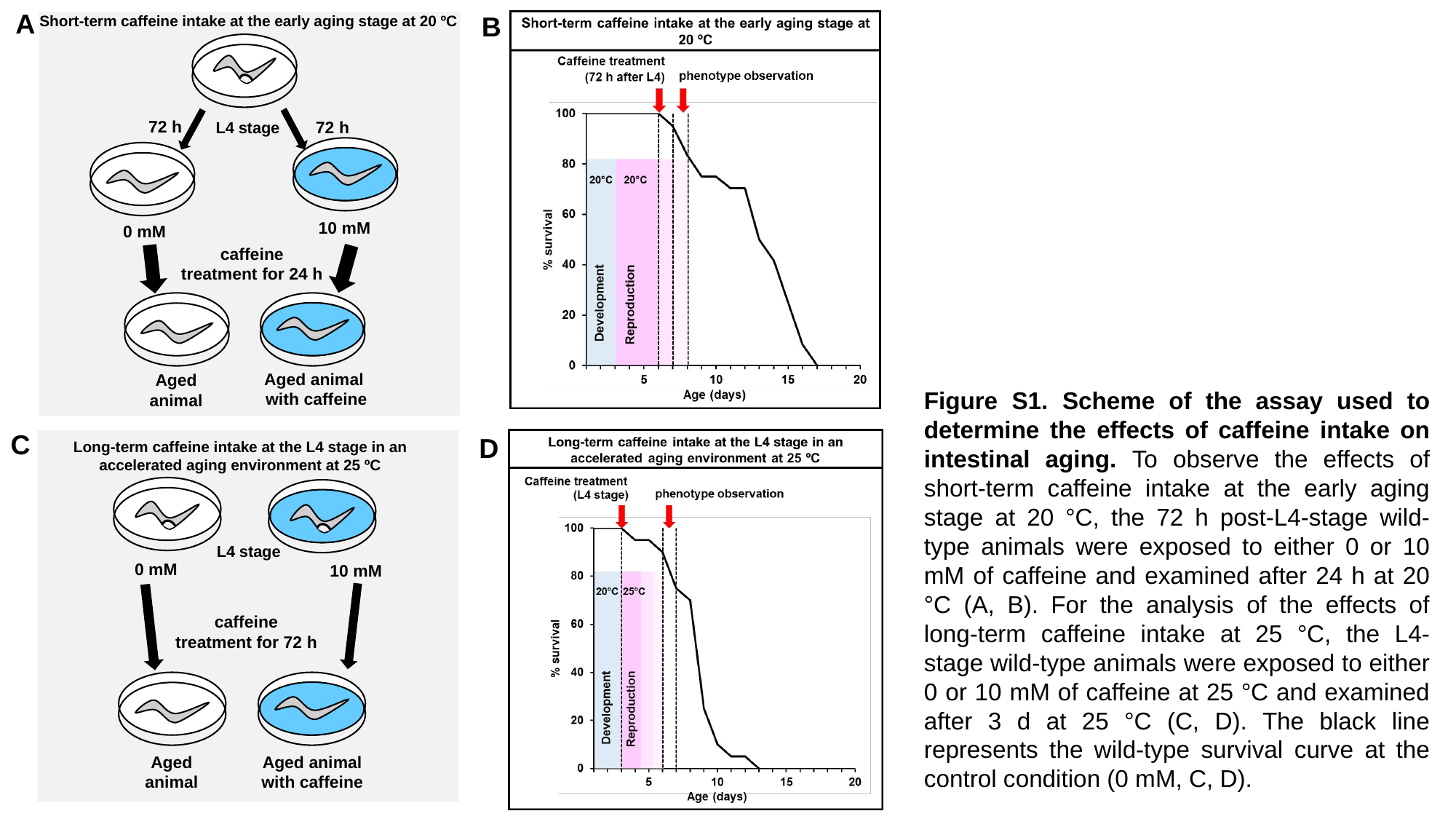

A
Short-term caffeine intake at the early aging stage at 20 ºC
72 h
72 h
L4 stage
10 mM
0 mM
caffeine
treatment for 24 h
Aged animal
with caffeine
Aged
animal
B
Figure S1. Scheme of the assay used to determine the effects of caffeine intake on intestinal aging. To observe the effects of short-term caffeine intake at the early aging stage at 20 °C, the 72 h post-L4-stage wild-type animals were exposed to either 0 or 10 mM of caffeine and examined after 24 h at 20 °C (A, B). For the analysis of the effects of long-term caffeine intake at 25 °C, the L4-stage wild-type animals were exposed to either 0 or 10 mM of caffeine at 25 °C and examined after 3 d at 25 °C (C, D). The black line represents the wild-type survival curve at the control condition (0 mM, C, D).
C
D
Long-term caffeine intake at the L4 stage in an accelerated aging environment at 25 ºC
L4 stage
0 mM
10 mM
caffeine
treatment for 72 h
Aged animal with caffeine
Aged
animal

## Slide 2
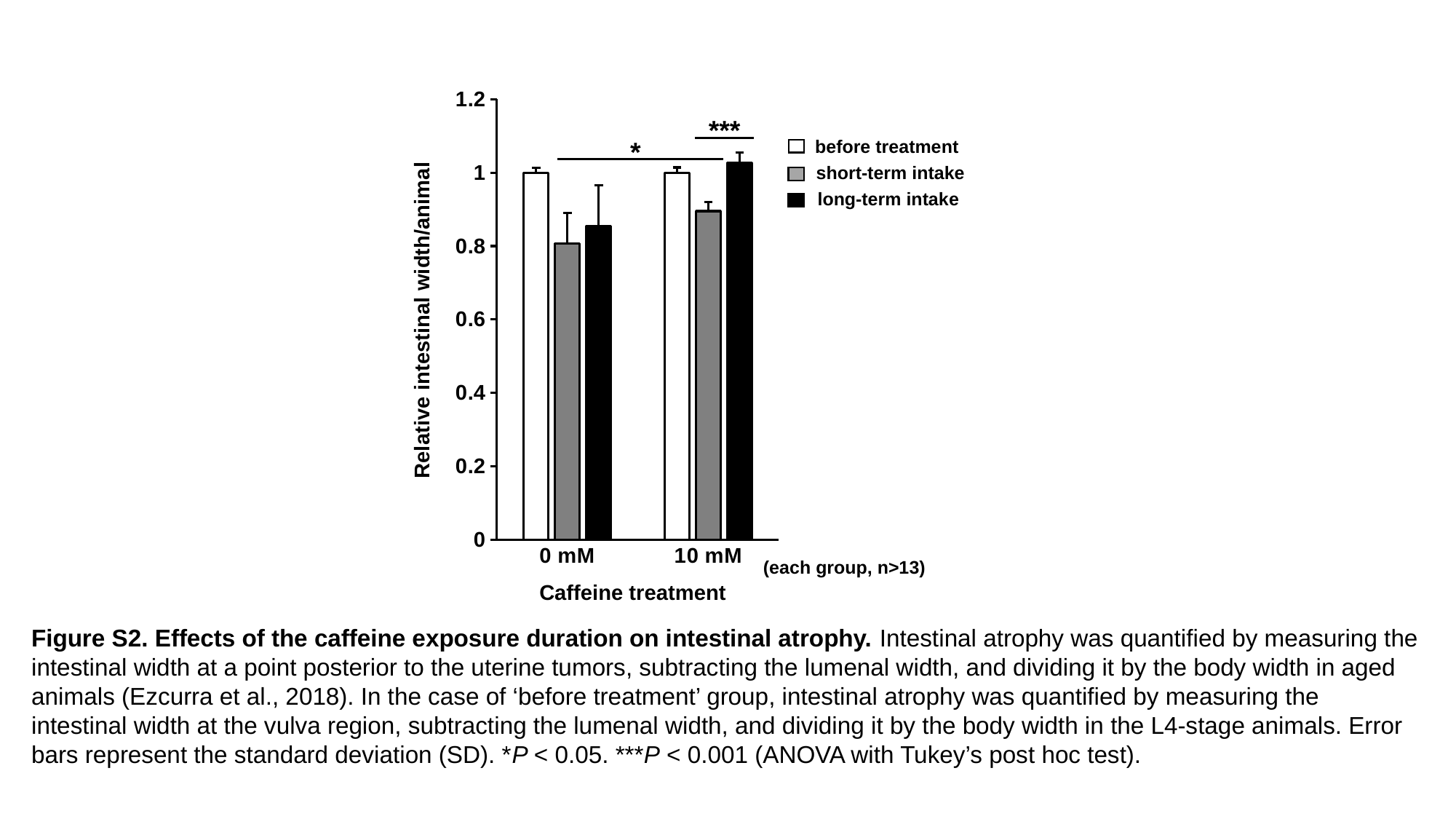

### Chart
| Category | before treatment | short-term intake | long-term intake |
|---|---|---|---|
| 0 mM | 1.0 | 0.80756515384615 | 0.8540146900145487 |
| 10 mM | 1.0 | 0.895288284615385 | 1.0277379656850014 |***
*
before treatment
short-term intake
long-term intake
Relative intestinal width/animal
(each group, n>13)
Caffeine treatment
Figure S2. Effects of the caffeine exposure duration on intestinal atrophy. Intestinal atrophy was quantified by measuring the intestinal width at a point posterior to the uterine tumors, subtracting the lumenal width, and dividing it by the body width in aged animals (Ezcurra et al., 2018). In the case of ‘before treatment’ group, intestinal atrophy was quantified by measuring the intestinal width at the vulva region, subtracting the lumenal width, and dividing it by the body width in the L4-stage animals. Error bars represent the standard deviation (SD). *P < 0.05. ***P < 0.001 (ANOVA with Tukey’s post hoc test).

## Slide 3
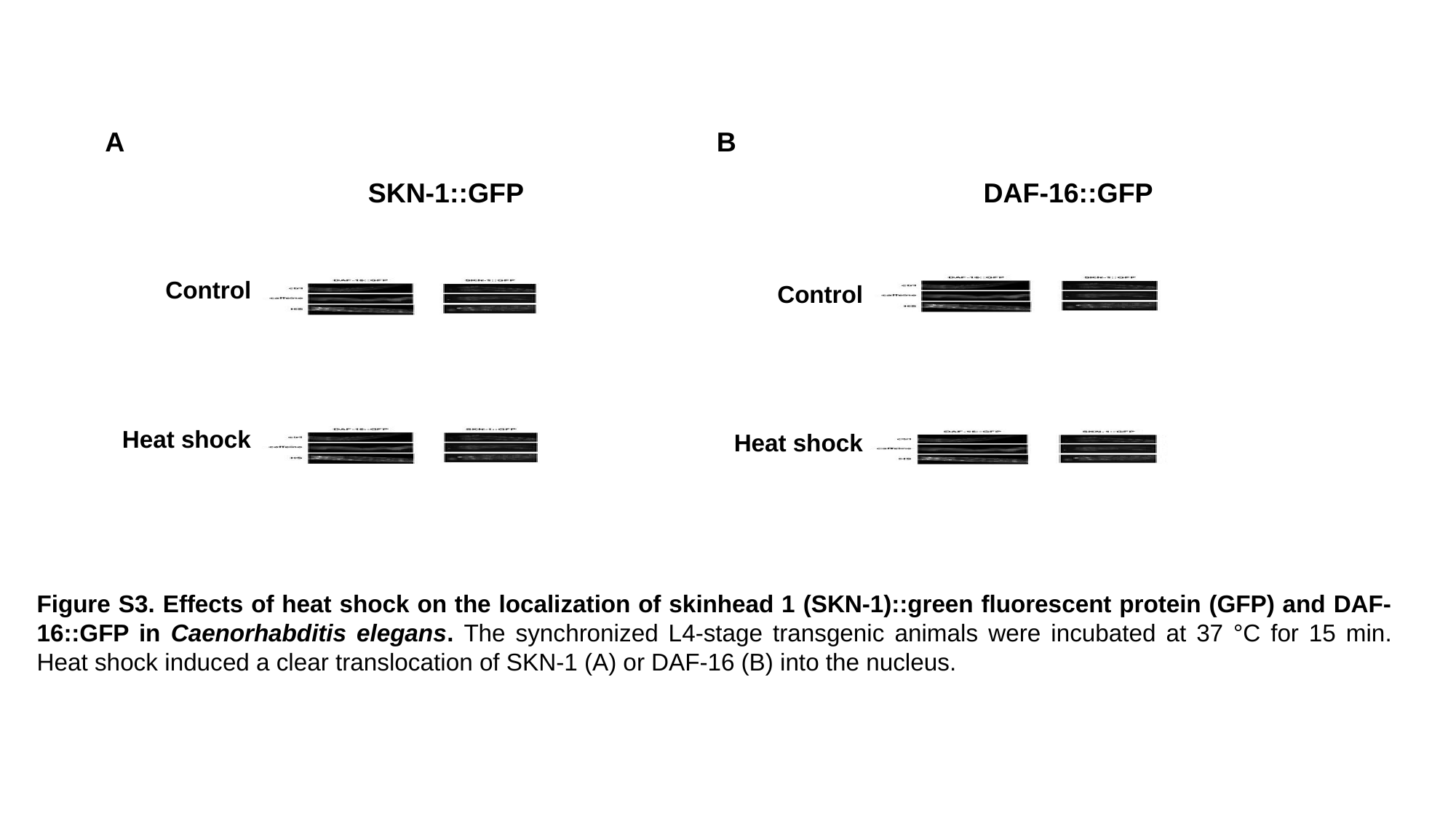

A
B
SKN-1::GFP
Control
Heat shock
DAF-16::GFP
Control
Heat shock
Figure S3. Effects of heat shock on the localization of skinhead 1 (SKN-1)::green fluorescent protein (GFP) and DAF-16::GFP in Caenorhabditis elegans. The synchronized L4-stage transgenic animals were incubated at 37 °C for 15 min. Heat shock induced a clear translocation of SKN-1 (A) or DAF-16 (B) into the nucleus.
